# Engaging Underserved Communities in COVID-19 Health Equity Implementation Research: An Analysis of Community Engagement Resource Needs and Costs

**DOI:** 10.3389/frhs.2022.850427

**Published:** 2022-03-17

**Authors:** Nicole A. Stadnick, Kelli L. Cain, Paul Watson, William Oswald, Marina Ibarra, Raphael Lagoc, Keith Pezzoli, Louise C. Laurent, Robert Tukey, Adrienn Borsika Rabin

**Affiliations:** 1 Department of Psychiatry, University of California, San Diego, La Jolla, CA, United States,; 2 University of California San Diego Altman Clinical and Translational Research Institute Dissemination and Implementation Science Center, La Jolla, CA, United States,; 3 Child and Adolescent Services Research Center, San Diego, CA, United States,; 4 Herbert Wertheim School of Public Health and Human Longevity Science, University of California, San Diego, La Jolla, CA, United States,; 5 The Global Action Research Center, San Diego, CA, United States,; 6 Department of Urban Studies and Planning, University of California, San Diego, La Jolla, CA, United States,; 7 Bioregional Center for Sustainability Science, Planning and Design, University of California, San Diego, La Jolla, CA, United States,; 8 Superfund Research Center, University of California, San Diego, La Jolla, CA, United States,; 9 Department of Obstetrics, Gynecology, and Reproductive Sciences, University of California, San Diego, San Diego, CA, United States,; 10 Department of Pharmacology, University of California, San Diego, San Diego, CA, United States

**Keywords:** community engagement, health equity, implementation, COVID-19, resources, costs

## Abstract

**Background:**

Meaningful community engagement is instrumental to effective implementation and sustainment of equitable public health interventions. Significant resources are necessary to ensure that community engagement takes place in culturally sensitive, trusted ways that optimize positive public health outcomes. However, the types and costs of resources best suited to enable meaningful community engagement in implementation research are not well-documented. This study’s objectives are (1) to describe a pragmatic method for systematically tracking and documenting resources utilized for community engagement activities, (2) report resources across phases of implementation research, and (3) provide recommendations for planning and budgeting for community engagement in health equity implementation research.

**Methods:**

Community engagement partners completed a tracking log of their person-hours for community engagement activities across three phases of community engagement (startup, early, maintenance) in two implementation research projects to promote equity in COVID-19 testing and vaccination for underserved communities. Both projects completed a six-session Theory of Change (i.e., a facilitated group discussion about current and desired conditions that culminated with a set of priorities for strategic change making) over 4 months with respective Community Advisory Boards (CAB) that included community organizers, promotores, federally qualified health center providers and administrators, and public health researchers. The reported person-hours that facilitated community member engagement were documented and summarized within and across project phases.

**Results:**

For both projects, the startup phase required the highest number of person-hours (*M* = 60), followed by the maintenance (*M* = 53) and early phase (*M* = 47). Within the startup phase, a total of 5 community engagement activities occurred with identifying and inviting CAB members incurring the greatest number of person-hours (*M* = 19). Within the early phase, a total of 11 community engagement activities occurred with coordinating and leading live interpretation (Spanish) during CAB sessions incurring the greatest number of person-hours (*M* = 10). The maintenance phase included 11 community engagement activities with time dedicated to written translation of CAB materials into Spanish incurring the greatest number of person-hours (*M* = 10).

**Conclusions:**

Study findings indicate that the most significant investment of resources is required in the startup period. Needed resources decreased, albeit with a greater diversity of activities, in later phases of community engagement with Spanish language translation requiring most in the later stage of the study. This study contributes to the community engagement and implementation science literature by providing a pragmatic tracking and measurement approach and recommendations for planning for and assessing costs to facilitate meaningful community engagement in public health implementation research.

## INTRODUCTION

Community engagement is now widely recognized as essential to public health and implementation science research and practice. The National Institute for Health Research (1) defines community engagement “as involving communities in decision-making and in the planning, design, governance and delivery of services; community engagement activities can take many forms, including service user networks, health-care forums, volunteering or interventions delivered by trained peers.” Extending to implementation science, community-engaged implementation research is characterized by implementation of evidence-based interventions within clinical or community settings using processes of community-engagement, inclusive but not exclusive to community-based participatory research (2). De Weger et al. (3) conducted a rapid realist review and identified eight action-oriented guiding principles for effective community engagement. The authors concluded that “meaningful participation” of citizens can only be achieved if organizational processes are adapted to ensure that they are inclusive, accessible and supportive of citizens (3).”

Engaging communities can be a lever for change coalescing a wider range of services across sectors, that are more tailored to the needs of the communities themselves and ultimately promoting improved community health and research quality (4, 5). There has been an evolving trend in policymaking from top-down approaches to stakeholder-engaged or participatory approaches to facilitate the likelihood that the intended outcomes (social, ecological, health) of the policy would be achieved (6). The need and value of community engagement has become more paramount during the COVID-19 pandemic era. For example, community members are urging for community engagement to build and sustain trust in health care, research, governmental and institutional systems among historically and currently underserved communities (7).

There have been attempts to systematically assess costs and cost-effectiveness associated with community engagement using quantitative, qualitative, and mixed methods (1, 8). Eisman et al. (9) emphasized the importance of considering cost from the perspective of multiple stakeholders, ranging from individual patients/participants to policy and economic representatives, when adopting an evidence-based practice. Anggraeni et al. (6) conducted a systematic review of the cost and value of stakeholder participation in policymaking. They developed a typology of the costs and benefits of stakeholder participation. In this typology, they categorized costs and benefits as tangible or intangible. Tangible costs and benefits may be travel costs, office supplies or consumable for meetings, access to appropriate technology, and payments for participation. Examples of intangible costs and benefits are opportunity cost of time, exclusion of intended stakeholders/beneficiaries, increased transparency in and shared decision-making, capacity-building/learning, and social cohesion. Challenges with costing exercises of community engagement include the retrospective and often inconsistently documented nature of community engagement activities, precise quantification of activities especially from multiple perspectives and time frames and identifying and measuring benefits. Anggraeni et al. (6) concluded that, “If the intent of participation is to give voice to the voiceless, lack of budgeting to enable the marginalized to participate may serve to do the opposite of what was intended – skew the policy in favor of the more powerful participants!”

Oliver et al. (10) caution about the negative costs associated with collaborative research, “co-production,” and community engagement. These include practical costs (e.g., administrative burden to arrange meetings, rooms and travel) personal costs (e.g., burnout and stress), professional costs to researchers, costs to research, costs to stakeholders, and costs to the research profession (e.g., credibility and utility of evidence questioned). The authors advise a thoughtful approach to co-production that involves “conscious and reflective research practice, evaluation of how coproduced research practices change outcomes, and exploration of the costs and benefits of coproduction.” Co-production is more likely successful when the primary purpose is to identify how best to implement a program or practice, the work cannot be carried out without the active cooperation of implementors and policymakers, and the time, resources, and expertise are available to engage key stakeholders throughout the appropriate points in the process.

While there are increasing applications of community-engaged implementation science to improve health equity [e.g., (11)], the resources that enable meaningful community engagement in implementation research from the perspectives of community members and partners are not well-known. Further, there are limited tools to pragmatically characterize the types of activities and required time commitment for successful community engagement. Through a case study design, this study’s objectives are to: (1) describe a pragmatic method for systematically documenting resources for community engagement activities, (2) report resources across phases of implementation research, and (3) provide recommendations for assessing and budgeting for community engagement in health equity implementation research. For this manuscript, we emphasize “tangible” costs and resources, in particular time-based activity reporting, based on the Anggraeni et al. (6) typology.

## METHODS

### Procedures

Four community partners from the Global Action Research Center (ARC) completed a documentation tracking log of their community engagement activities during the Theory of Change development across two research projects (described below). The Global ARC is a non-profit, social change organization that partners with academic institutions and community organizations to facilitate community-engaged environmental justice and health equity projects. The Global ARC partnered with the University of California San Diego to co-lead the community engagement activities within both research projects.

The documentation tracking log was a simple matrix developed in a word editing program. It included rows for each community engagement activity that occurred in each of three phases of the Theory of Change process. Community engagement activities were identified using an iterative approach where the research team identified an initial set of activities that were confirmed and refined through feedback from the community partners. Activities were organized across three phases: (1) startup: five activities prior to the first Theory of Change session, (2) early: 11 activities that occurred during and between the first and second Theory of Change sessions, (3) maintenance: 11 activities that occurred during and between the third, fourth, and fifth Theory of Change sessions. See Table 1 for a description of each activity. The community engagement activities tracked within each phase were identified in an iterative manner based on the project management timeline and weekly group discussions between the Global ARC and university research partners. Each of the four Global ARC partners reported the average number of hours spent weekly on each community engagement activity within each phase.

The four community partners who completed the community engagement resource tracking log were: (1) the CEO and President of the Global ARC, (2) a PhD-level Director at the Global ARC, (3) a bachelor’s-level Bilingual (Spanish/English) Community Outreach Specialist and (4) a bachelor’s level Technology Outreach Specialist. These partners completed the resource tracking log within 1 month of completing the six Theory of Change sessions.

The two research projects from which these community engagement data were drawn are described in the following sections. Both projects were approved by the University of California San Diego Institutional Review Board. The first research project is: Community-driven Optimization of COVID-19 testing to Reach and Engage underserved Areas for Testing Equity (CO-CREATE). CO-CREATE is a 2-year study funded through the NIH RADx for Underserved Population initiative. The key objective is to understand practices, barriers, and facilitators to access and uptake of COVID-19 testing and follow-up for underserved community members from the perspectives of patients, providers, and organizational leaders at a federally qualified health center with clinics in South San Diego near the US/Mexico border.

The second research project is: Share, Trust, Organize, Partner: The COVID-19 California Alliance (STOP COVID-19 CA). The STOP COVID-19 CA project is part of the NIH Community Engagement Alliance (CEAL) Against COVID-19 Disparities. The CEAL program includes community-academic teams in 11 states throughout the US and focuses on COVID-19 awareness and education research, especially among Black, Latino, Indigenous, refugee and immigrant populations. The California CEAL team is locally known as STOP COVID-19-CA and involves a network of 11 institutions in California, including UC San Diego. The UC San Diego CEAL project conducted rapid community engagement to assess multi-level barriers, facilitators, and processes to engaging individuals from underserved communities in COVID-19 screening and vaccine trials as well as to advance vaccine uptake.

Both CO-CREATE and STOP COVID-19 CA established a Community Advisory Board (CAB) to engage in developing a Theory of Change that guided each project’s overarching aims. Table 2 describes the composition of each CAB that included community health workers, community leaders, healthcare administrators and providers, public health researchers, and policymakers. Both CABs included Spanish-speaking members so there was concurrent live Spanish-English and English-Spanish interpretation. The CABs met virtually monthly in the early evenings for 2 hours. Each CAB member received compensation in the form of a $100 honorarium that was mailed after each meeting.

The Theory of Change process used in these research projects is described in detail in Stadnick et al. (12). In brief, Theory of Change is an extended, multiple session, highly interactive and participatory consensus-building process. The participants collectively identify (1) their desired outcome, (2) the barriers and constraints that stand in the way of realizing that outcome, and (3) interventions, that if implemented, can reduce conditions that thwart progress and thereby help move the group toward it desired outcome. A good Theory of Change can improve planning, implementation and evaluation of public health programs and yield a comprehensive approach/pathway to realizing desired outcomes in particular contexts. A project-specific Theory of Change was developed to identify the necessary conditions, actions, and measures of success needed to reduce disparities in access to and benefit from COVID-19 testing, vaccination, and participation in clinical trials.

### Data Analysis

The resource tracking logs completed by each community partner served as the primary data source for this report. The reported person hours for each community engagement activity were descriptively summarized in the following ways: (1) a summed aggregate across community partners by project phase (startup, early maintenance) and within project phase, (2) a proportional aggregate across community partners by project phase (startup, early maintenance) and within project phase,(3) a sum of hours of each community partner by phase and within phase. In addition, we included the costs for tangible resources to support participation of community members in the CABs. Specifically, we calculated the costs for one-time technology supports (i.e., hot-spot devices and tablets) and recurring meeting stipends provided to CAB members.

## RESULTS

**FIGURE 1 |:**
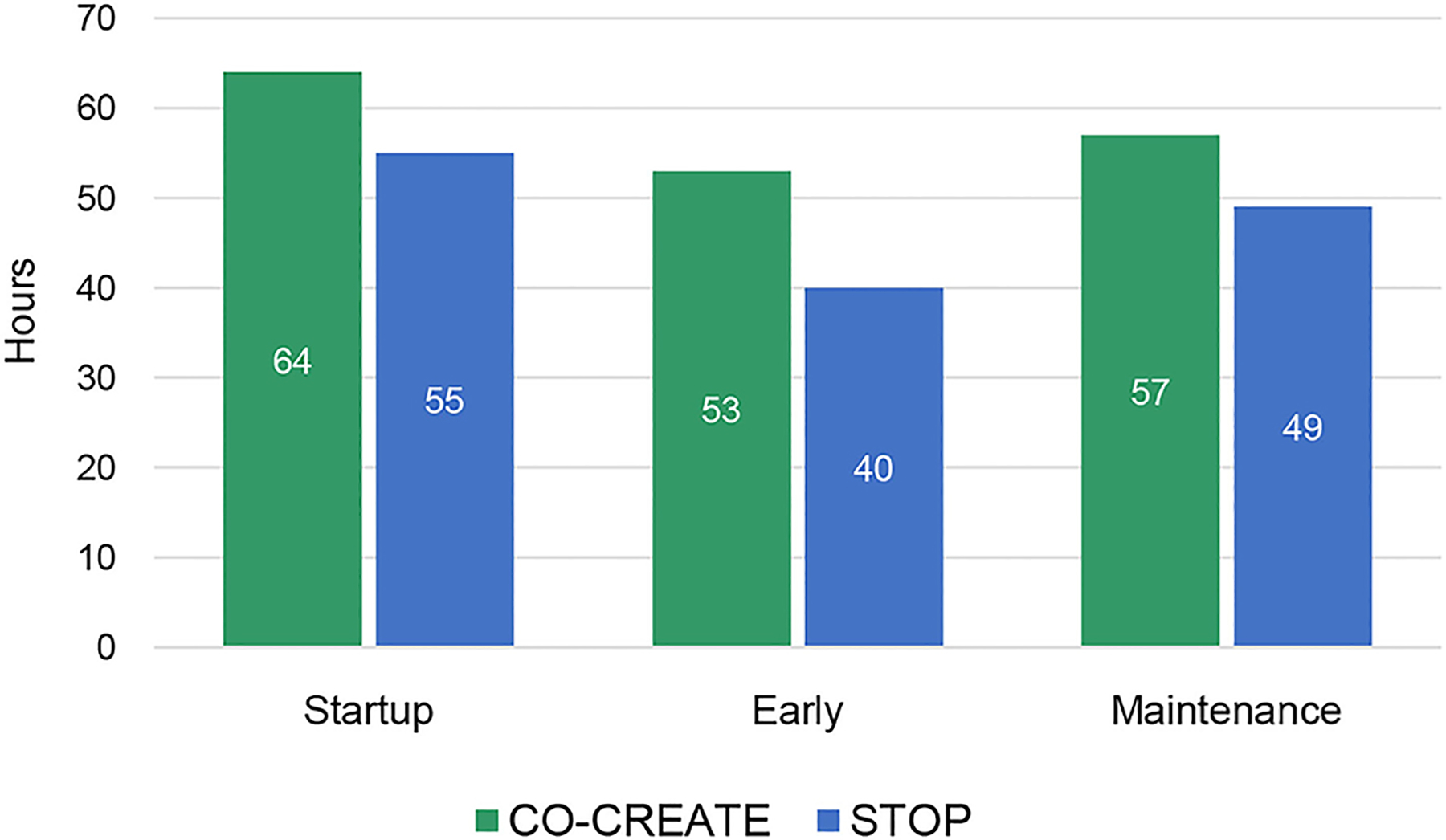
Total community engagement hours across phases for CO-CREATE and STOP COVID-19 CA.

**FIGURE 2 |:**
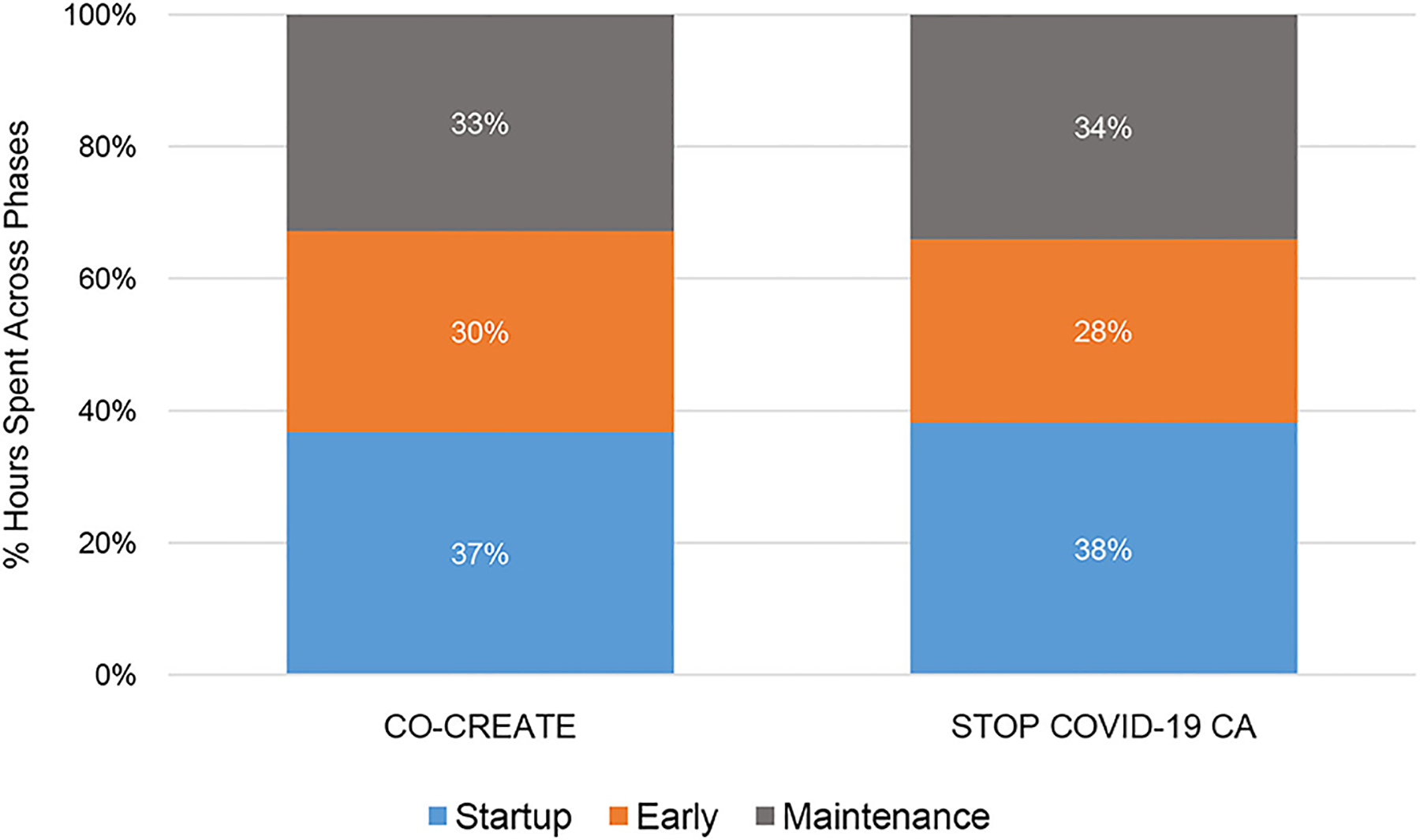
Proportion of community engagement hours across phases for CO-CREATE and STOP COVID-19 CA.

Overall, CO-CREATE required more person hours than STOP across startup, early, and maintenance phases of the Theory of Change process. For both projects, the startup phase required the highest number of person hours, followed by the maintenance phase, and then the early phase. This finding is also borne out in the proportional data although the proportions of time spent within each phase and across both projects was roughly equivalent. For example, 37% of documented hours was dedicated to the startup phase in CO-CREATE and 38% of documented hours was dedicated to the startup phase in STOP. Please see Figures 1, 2.

At the individual-level, the CEO/Director 1 reported the highest number of community engagement hours across phases and projects. For example, the CEO/Director 1 reported 24 and 16 weekly hours during the startup phase (CO-CREATE and STOP, respectively).

**FIGURE 3 |:**
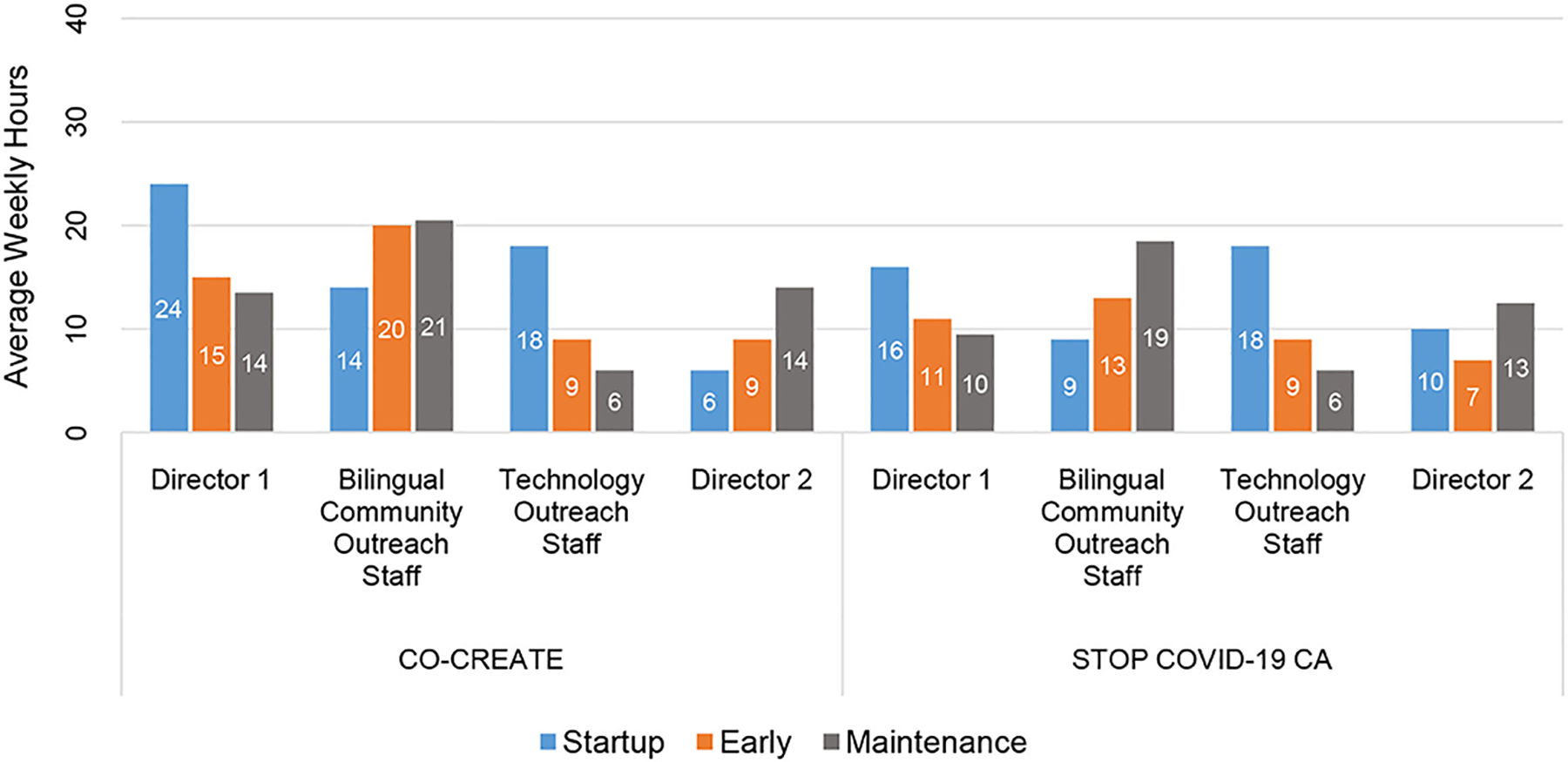
Total average weekly hours per community partner by phase for CO-CREATE and STOP COVID-19 CA.

In the startup phase, the Technology Outreach Specialist reported the second highest number of weekly community engagement hours (18 h). In the early and maintenance phases, the Bilingual Community Outreach Specialist reported the highest number of weekly community engagement hours (range = 13–20.5 h). Please see Figure 3.

**FIGURE 4 |:**
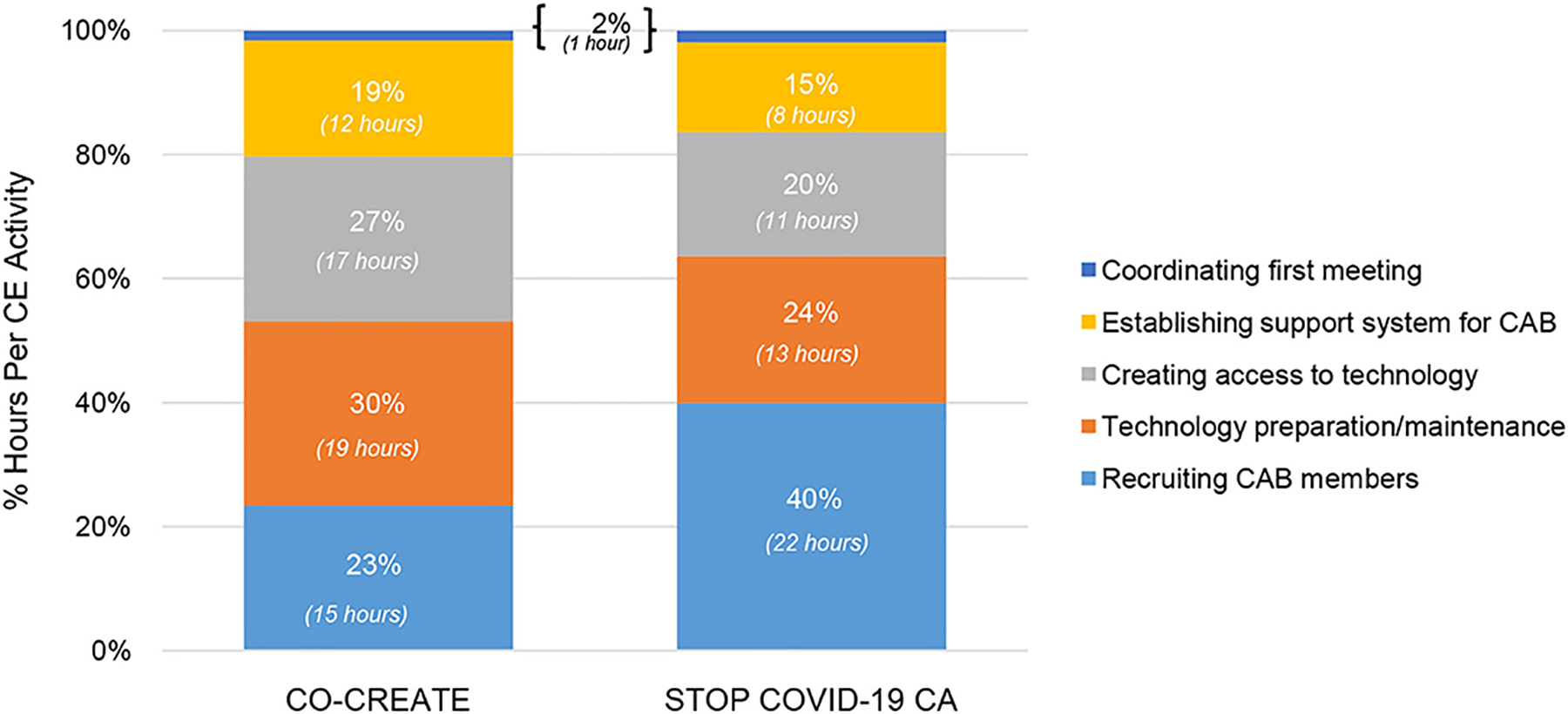
Proportion of community engagement hours per activity during startup for CO-CREATE and STOP COVID-19 CA.

In the startup period, the community engagement activities that required the highest number of person hours were identifying and inviting CAB members, technology preparation and maintenance, and creating access to technology devices and software for CAB members. A total of 5 community engagement activities occurred in this phase. The proportional differences in time spent on recruiting CAB members (23% for CO-CREATE vs. 40% for STOP COVID-19 CA) between the projects is likely due to the different composition of CAB members. Specifically, for STOP COVID-19 CA, the goal was to recruit community members or organizers who were from specific and unique African American, immigrant, and refugee communities. This required meeting with more individuals to determine the best fit and experiences to meaningfully engage with the CAB. In contrast, less time was spent on creating and maintaining access to technology devices and software for the STOP COVID-19 CA CAB members (24% and 20% for STOP COVID-19 CA vs. 30 and 27% for CO-CREATE) because the majority were working professionals in their community who generally had high levels of technology literacy. Specific costs that were incurred during this phase were for purchasing hot-spot devices and tablets for a subset of community members who needed reliable access to participate in the virtual CAB meetings. A total of ~$500 per person was spent on procuring technology supports (laptops and internet hot spots) for four community members. Please see Figure 4.

**FIGURE 5 |:**
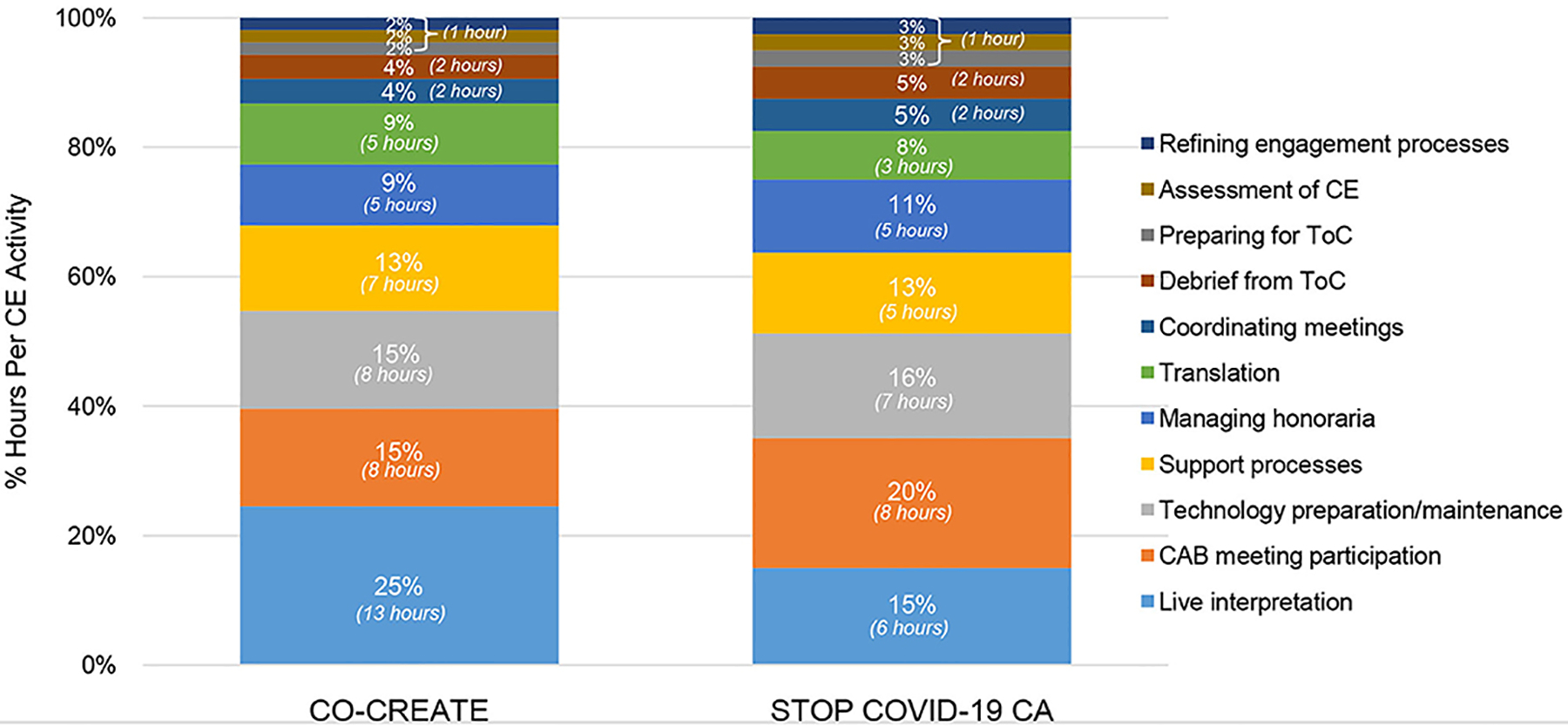
Proportion of community engagement hours per activity during the early phase for CO-CREATE and STOP COVID-19 CA. ToC, Theory of Change; CE, community engagement.

In the early phase, the community engagement activities that required the highest number of person hours were: live interpretation and troubleshooting, participating in the CAB meetings, technology preparation and maintenance, and establishing support processes for the CAB members in between Theory of Change sessions. A total of 11 community engagement activities occurred in this phase. The primary difference between the two projects was in the proportion of time spent on live interpretation execution and troubleshooting with interpreter staff (25% for CO-CREATE vs. 15% for STOP COVID-19 CA). This can largely be explained by the different CAB compositions. For CO-CREATE, there were nine CAB members (out of 22) who preferred Spanish as their primary language. A greater amount of time was needed within the CAB meetings to pause and ensure high quality interpretation. In addition, additional time was needed outside of the CAB meetings for meetings between the professional interpreters and Global ARC staff to refine interpretation practices during live meetings. Specific costs incurred during this phase were the stipends offered to community members for their CAB participation in the first two meetings. Each CAB member was offered a $100 stipend per CAB meeting. This amount totaled to $6600 across both projects for stipend purchase. Please see Figure 5.

**FIGURE 6 |:**
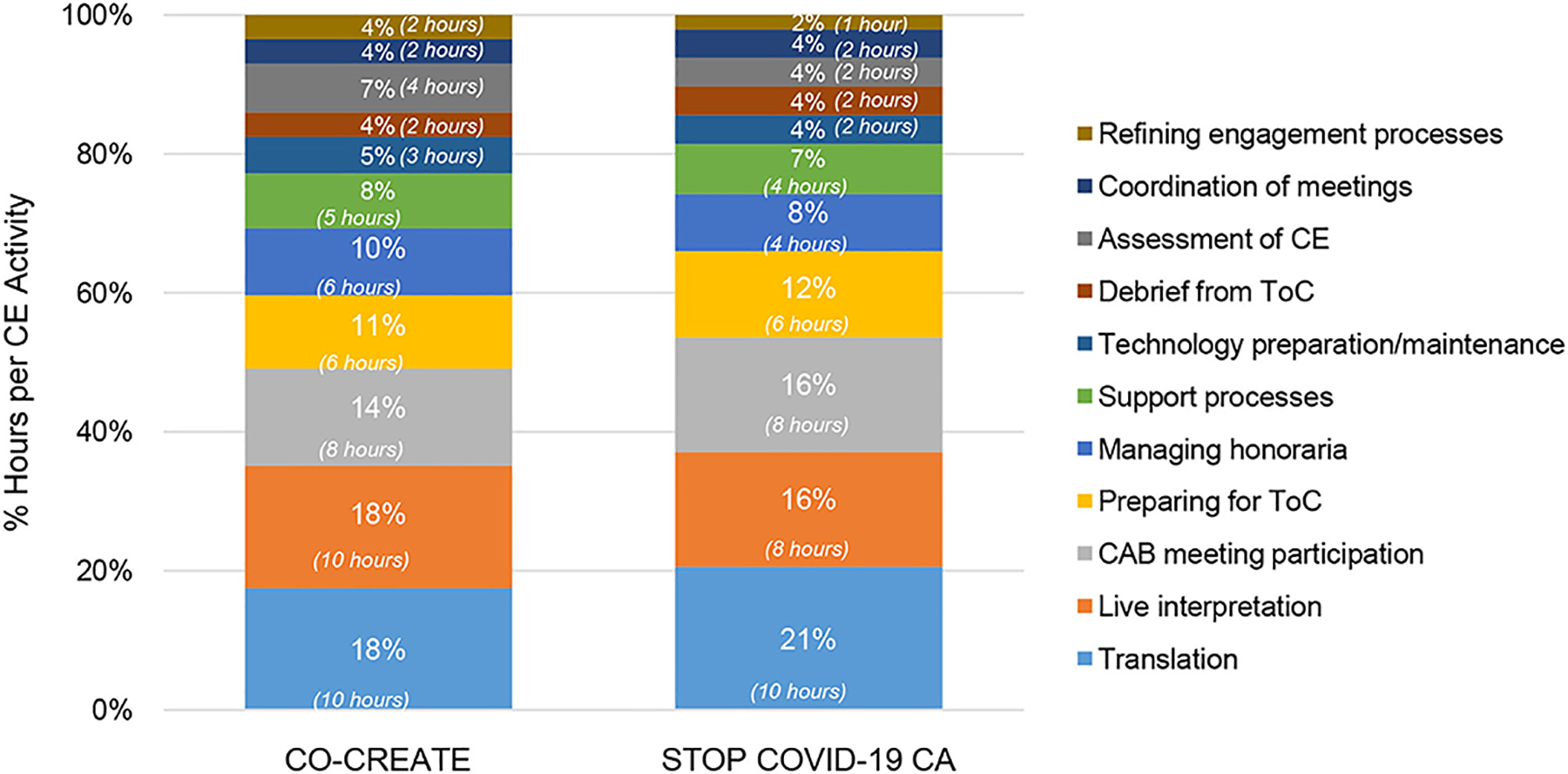
Proportion of community engagement hours per activity during the maintenance phase for CO-CREATE and STOP COVID-19 CA. ToC, Theory of Change; CE, community engagement.

In the maintenance phase, the community engagement activities that required the highest number of person hours were written translation, live interpretation and troubleshooting, participating in the CAB meetings, and preparing for the Theory of Change sessions. A total of 11 community engagement activities occurred in this phase. There were no major differences noted in community engagement time spent between the two projects. Notably, the difference in time spent on live interpretation and troubleshooting decreased during the maintenance phase (18% for CO-CREATE vs. 17% for STOP COVID-19 CA), suggesting a positive impact on the refinement practices identified and implemented during the early phase. Specific costs incurred during maintenance phase continued to be the stipends offered to community members for their CAB participation in the last four meetings. This amount totaled to $26,400 across both projects for stipend purchase. Please see Figure 6.

## DISCUSSION

This report provides a pragmatic methodology for characterizing the types of activities and associated time commitment (resources) for authentic community engagement in health equity implementation research. We illustrated application of this methodology to two COVID-19 health equity projects completing a CAB-driven Theory of Change. While costs, resources, and benefits can be calculated and categorized in many ways (1, 8, 9), we focused our assessment on tangible costs and resources (6) vis-à-vis time-based activity reporting from the perspectives of community partners and expenditures for technology equipment to facilitate community participation.

Through descriptive analysis, we identified that the community engagement activities in the startup phase required the greatest number of person-hours compared to the early and maintenance phases of the projects. Specifically, across four community partners (two directors and two staff) who co-led these projects, a total of 64 and 55 h weekly were dedicated to community engagement activities for CO-CREATE and STOP COVID-19 CA, respectively. In addition, the startup period required the fewest number of discrete activities (5 vs. 11 in the other phases), but more concentrated time was spent on these activities. The Global ARC director and the bilingual community outreach staff member reported the greatest number of hours spent on community engagement activities across phases and projects.

A few differences between the two projects were noted in total and in the startup and early phases. Overall, CO-CREATE required a higher number of person-hours in each phase to facilitate community engagement activities compared to STOP COVID-19 CA. This finding is most likely due to the size and composition of the CO-CREATE CAB. That is, the CO-CREATE CAB comprised 22 members with distinct professional roles and expertise: promotores who generally preferred Spanish as their primary language, public health researchers, healthcare providers, and healthcare administrators. In contrast, the STOP COVID-19 CA CAB comprised 11 members all of whom were community leaders, albeit from different ethnic and cultural communities, and largely comfortable communicating in English. In addition, most of the STOP COVID-19 CA CAB members had well-established collaborative relationships with the Global ARC through previous or concurrent community engagement work. The strength of these positive working relationships likely also impacted the lower number of reported person-hours dedicated to community engagement activities for the STOP COVID-19 CA project.

Specific to project differences across phases, more time was spent during startup on identifying and recruiting CAB members but less time on technology access activities for STOP COVID-19 CA compared to CO-CREATE. In the early phase, more time was spent on live interpretation and interpreter quality assurance for CO-CREATE compared to STOP COVID-19 CA. During the maintenance phase, the proportion of time spent on each community engagement activity was very similar, within ≤4% for each activity between projects. Project differences within these phases is also likely driven by the composition and characteristics of each project’s CAB such as size, language preference, and professional and lived experiences and expertise.

While our specific analysis and results were centered on CAB development of Theories of Change and do not represent all types of community engagement work, learnings from our application of this resource assessment method suggest several recommendations for others pursuing community engagement in health equity implementation research. First, it is critical to consider resources needed for meaningful stakeholder engagement broadly and early in the planning of public health and health services research projects (i.e., proposal writing stage) and account for these costs in the budget. In line with guiding principles for meaningful community engagement (3, 6), resources should be considered for time estimates for diverse personnel for the initial (start-up) an ongoing engagement of community participants, the costs associated with technology and devices, costs associated with language accommodations and honoraria for participants. These estimations must be made in collaboration with community partners and reflected in the budget estimates for the research proposal. Relatedly, resources needed for successful stakeholder engagement will likely vary across the project which should be reflected in budget estimates. We note that while there were costs incurred related to the virtual methods of CAB interaction, there are also costs that were not incurred and that may need to be considered for non-virtual or hybrid (virtual and in-person) interactions. These might include food/beverage, childcare, and transportation (public transportation vouchers, gas cards).

Second, language accommodations are a critical ingredient of engaging all but especially underserved communities. These accommodations have substantial cost implications on multiple levels including preparation and distribution of materials for meetings, live interpretation during meetings, translation of materials to English after meetings, and ensuring that data collection instruments are available in multiple languages based on community participant preferences.

Third, if community engagement happens virtually—as it did in many cases during the pandemic—there is a critical need to consider what costs will be accumulated through the technology accommodations to support community engagement. These can include providing devices and hot spots to allow for connection, providing technical assistance to participants before and during meetings. Time spent on coordinating with community participants outside of meetings should not be underestimated and can require substantial amount of staff time especially in the startup phase of the project. Finally, in line with the call from Eisman et al. (9) to consider costs of implementation from multiple stakeholder perspectives, it can be beneficial to develop a pragmatic approach to documenting costs associated with engaging community partners developing and using a template (such as the one we provided in Supplementary Materials). Since key resource categories will vary across projects, templates should be adapted iteratively in collaboration with community partners.

This methodology and case example findings are a necessary but insufficient step toward making the often invisible practice of meaningful community engagement visible. Importantly, while there are tangible costs and resources required for community engagement, there are also tangible and intangible benefits (positive externalities) of this type of work that might not be otherwise possible. These positive externalities might include fortifying relationships and trust between academic and community members that may facilitate more expedient responses to future public health crises or concerns; developing equitable public health programs with true sustaining power because they fit with the realities and strengths of real-world communities.

A key limitation of our study and important next step is to expand the documentation of cost by including expenses related to salaries of research and community partner team members as well as time for community member participation. Doing so will allow for a natural next step that allows for comparing community engagement resource needs across a wider range of community engagement efforts (beyond that of CAB development of Theories of Change). Additionally, a future direction is to develop methods to assess the costs of abstract but fundamental community engagement activities to build strong civic infrastructure. These activities may include the institutional groundwork, protocols, ethics, and rules of engagement to enable trusted, ongoing, co-evolutionary bidirectional learning capacity for a particular project and the public good. The priorities of specific partners will naturally change over time. Community engagement as embedded or expected in implementation science and action research, may facilitate intentional consideration of the tangible and abstract costs needed for meaningful community engagement to promote equity in health outcomes and in community participation in the scientific and implementation enterprise.

## Supplementary Material

Suppl Material - Table 1

## Figures and Tables

**TABLE 1 |:** Community engagement activities by phase.

Activity	Startup phase	Early phase	Maintenance phase
Identifying and inviting CAB members	X		
Creating access to technology	X		
Technology preparation and maintenance	X	X	X
Scheduling and coordinating meetings	X	X	X
Establishing CAB support systems	X	X	X
Translation (written)		X	X
Interpretation (live)		X	X
Preparing content for Theory of Change sessions		X	X
Participating in CAB meetings		X	X
Debriefing from Theory of Change sessions		X	X
Assessment of community engagement		X	X
Refining engagement processes		X	X
Managing CAB honoraria		X	X

**TABLE 2 |:** Community Advisory Boards for CO-CREATE and UC San Diego STOP COVID-19 CA.

CO-CREATE	STOP COVID-19 CA
* **9 Community partners** Promotores Coalition Latinos y Latinas en Accion	**11 Community leaders** *Comite Organizador Latinos de City Heights Karen Organization of San Diego Kupanda Kids Partnership for the Advancement of New Americans Refugee Health Unit/Center for Community Health Somali Bantu Community South Sudanese Community Center The Humanity Movement Unity in the Community YouthWill
**6 Public health research partners** University of California San Diego San Diego State University Loma Linda University	**2 Policy partners (non-voting CAB members)** San Diego City Council, District 9, Community Empowerment
**7 Clinic partners** Providers Administrators	

* Spanish was their preferred language used in CAB meetings.

## Data Availability

The original contributions presented in the study are included in the article/Supplementary Material, further inquiries can be directed to the corresponding author/s.
