# Corrigendum: Engaging underserved communities in COVID-19 health equity implementation research: an analysis of community engagement resource needs and costs

**DOI:** 10.3389/frhs.2025.1632312

**Published:** 2025-06-03

**Authors:** Nicole A. Stadnick, Kelli L. Cain, Paul Watson, William Oswald, Marina Ibarra, Raphael Lagoc, Keith Pezzoli, Louise C. Laurent, Robert Tukey, Adrienn Borsika Rabin

**Affiliations:** ^1^ Department of Psychiatry, University of California, San Diego, La Jolla, CA, United States; ^2^ University of California San Diego Altman Clinical and Translational Research Institute Dissemination and Implementation Science Center, La Jolla, CA, United States; ^3^ Child and Adolescent Services Research Center, San Diego, CA, United States; ^4^ Herbert Wertheim School of Public Health and Human Longevity Science, University of California, San Diego, La Jolla, CA, United States; ^5^ The Global Action Research Center, San Diego, CA, United States; ^6^ Department of Urban Studies and Planning, University of California, San Diego, La Jolla, CA, United States; ^7^ Bioregional Center for Sustainability Science, Planning and Design, University of California, San Diego, La Jolla, CA, United States; ^8^ Superfund Research Center, University of California, San Diego, La Jolla, CA, United States; ^9^ Department of Obstetrics, Gynecology, and Reproductive Sciences, University of California, San Diego, San Diego, CA, United States; ^10^ Department of Pharmacology, University of California, San Diego, San Diego, CA, United States

**Keywords:** community engagement, health equity, implementation, COVID-19, resources, costs

A Corrigendum on Engaging underserved communities in COVID-19 health equity implementation research: an analysis of community engagement resource needs and costs By Stadnick NA, Cain KL, Watson P, Oswald W, Ibarra M, Lagoc R, Pezzoli K, Laurent LC, Tukey R, Rabin AB (2022). Front. Health Serv. 2:850427. doi: 10.3389/frhs.2022.850427

In the published article, there was an error in the Funding statement. The authors wish to revise the Funding section of the original article as part of the funding acknowledgment (the National Institutes of Health (NIH) Agreement OT2HL158287) was inadvertently omitted.

The correct funding statement appears below.

**Funding**

This work was funded with support from the National Institutes of Health: P42 ES010337-19S2 RADx-UP Supplement (LL and RT); OTA-21-312-0217571-66106l (AR and NS); K23 MH110602 (NS); R34 MH120190 (NS), and the UC San Diego ACTRI Dissemination and Implementation Science Center (AR and NS). This research was, in part, funded by the National Institutes of Health (NIH) Agreement OT2HL158287. The views and conclusions contained in this document are those of the authors and should not be interpreted as representing the official policies, either expressed or implied, of the NIH.

The authors apologize for this error and state that this does not change the scientific conclusions of the article in any way. The original article has been updated.

