# Supplementary material for: Engaging Underserved Communities in COVID-19 Health Equity Implementation Research: An Analysis of Community Engagement Resource Needs and Costs
Source: Front Health Serv. 2022 Mar 17;2:850427. doi: 10.3389/frhs.2022.850427 (PMC9574473; doi:10.3389/frhs.2022.850427)
Supplement: Supplementary file 1 [file Table_1.docx]

**Supplementary Material**

**Community Engagement Resources Tracking**

*Instructions:* Use this tracking sheet to document the weekly hours spent on community engagement activities or cost of items to support community engagement activities. We recommend requesting that each community partner who was involved in community engagement efforts complete a tracking sheet. We divided reporting into three phases (start-up, early, maintenance) but these phases may need to be altered based on the specifics of your project.

**Community Partner Reporter (Name): _________________________________**

| ***Phase 1: Start Up*** | | |
| --- | --- | --- |
| **Community Engagement Activity or Item** (e.g., recruiting community members, debriefing from community engagement meetings, procuring technology equipment) | **Weekly Hours on Project** | **Cost/Resources** (e.g., staff time, technology equipment, honoraria) |
|  | XX hours—reporter |  |
|  |  |  |
| ***Phase 2: Early*** | | |
| **Community Engagement Activity or Item** | **Weekly Hours on Project** | **Cost/Resources** |
|  |  |  |
|  |  |  |
| ***Phase 3: Maintenance*** | | |
| **Community Engagement Activity or Item** | **Weekly Hours on Project** | **Cost/Resources** |
|  |  |  |
|  |  |  |
